# Large-scale docking approaches to the kinome

**DOI:** 10.1186/1758-2946-6-S1-P31

**Published:** 2014-03-11

**Authors:** Denis Schmidt, Peter Kolb

**Affiliations:** 1 Pharmaceutical Chemistry, Philipps-University, Marburg, Germany

## 

Docking nowadays is a widely used tool and has successfully and repeatedly been applied to identify hits with new chemical scaffolds [1]. Despite its successful applications, docking still suffers mainly form its scoring functions, which tend to be optimized for speed to the disadvantage of accuracy. Because of that, docking successfully enriches active molecules compared to inactive ones, however the false positive rate in docking runs is still very high. Several attempts have been made to improve docking by applying consensus scoring approaches using multiple structures or softwares or by rescoring compounds using normalization procedures [2-4]. At the same time, the number of large datasets of activity data derived from high-throughput screening methods has strongly increased during the last years, especially for kinases, allowing re-evaluation of these techniques [5-7]. We herein carried out a large-scale docking approach using 650 different kinase crystal structures. To the best of our knowledge, this is the largest number of targets docked to at the same time. Subsequently, different normalization techniques were re-evaluated using the experimental activity data as target function. Furthermore, we investigated the similarity of the kinase structures within the "docking universe" and correlated these similarities with other similarity metrices.
